# Evaluation of the effect of reduced-dose pneumococcal conjugate vaccine schedules on vaccine serotype carriage in children and their caretakers in a naïve population in Vietnam: Protocol for a cluster randomized non-inferiority trial

**DOI:** 10.12688/gatesopenres.14742.1

**Published:** 2023-07-20

**Authors:** Lay-Myint Yoshida, Stefan Flasche, Kim Mulholland, Hien-Anh Nguyen, Cattram Nguyen, Michiko Toizumi, Duc-Anh Dang

**Affiliations:** 1 Department of Pediatric Infectious Diseases, Institute of Tropical Medicine, Nagasaki University, Nagasaki, Japan; 2 Centre For Mathematical Modelling of Infectious Diseases, London School of Hygiene and Tropical Medicine, London, UK; 3 Department of Infectious Disease Epidemiology, London School of Hygiene and Tropical Medicine, London, UK; 4 Department of Infection and Immunity, Murdoch Children's Research Institute, Parkville, Victoria, Australia; 5 Department of Bacteriology, National Institute of Hygiene and Epidemiology, Hanoi, Vietnam

**Keywords:** Pneumococcal conjugate vaccine (PCV), Reduced dosing schedule, Vietnam, vaccine trial

## Abstract

**Introduction: **The WHO currently recommends giving pneumococcal conjugate vaccines (PCVs) as three doses – either three doses in infancy with Pentavalent vaccine (3p+0), or two doses in infancy followed by a booster around 12 months (2p+1). However, their high price is a barrier to introduction and sustainability in low and middle-income countries. We hypothesize that a schedule with a single priming and a booster dose (1p+1) may maintain similar levels of protection for the community by sustaining herd immunity, once circulation of vaccine types has been controlled.

**Methods and analysis: **We will conduct a cluster randomized trial with four intervention arms (1p+1, 0p+1, 2p+1, 3p+0) and three unvaccinated clusters in the 27 communes of Nha Trang, central Vietnam. A PCV catch-up vaccination campaign to all children under three years of age will be performed at the start of the trial. The primary endpoint is non-inferiority of the1p+1 schedule if compared to the WHO standard 2p+1 and 3p+0 schedules in reducing vaccine serotype carriage prevalence in infants. We will also explore impact of 0p+1 schedule. A baseline and annual pneumococcal carriage surveys of 6480 participants per survey covering infants, toddlers and their mothers will be conducted.

**Ethics and dissemination:** Ethical approvals were obtained from the ethical review committees of Institute of Tropical Medicine, Nagasaki University (151203149-2) and the Ministry of Health, Vietnam (1915/QD-BYT). The results, interpretation and conclusions will be presented at national and international conferences, and published in peer-reviewed open access journals.

**Trial registration number: **NCT02961231

## Introduction

Before the widespread use of pneumococcal conjugate vaccines (PCVs) *Streptococcus pneumoniae* was associated with about 14.5 million episodes of serious pneumococcal disease and more than 800,000 deaths globally in children younger than five years ^1^, with the highest burden among low and middle income countries ^2^. While PCVs have substantially reduced the burden of pneumococcal disease, their high price has delayed PCV introduction among middle income countries without financial aid, and will make it difficult for low income countries that have introduced PCVs with the help of the Gavi, the Vaccine Alliance, to sustain their programs once they transition from Gavi support ^3^.

WHO currently recommends giving three PCVs doses – either three doses in infancy (3p+0), or two doses in infancy followed by a booster around the end of the first year of life (2p+1) ^4^. The 3p+0 schedule is used in most low-income countries introducing PCV with Gavi support and aims to provide maximum direct protection early in life where young children are most susceptible to pneumococcal disease. However, almost all high-income countries are using a booster dose schedule that in addition aims to prevent transmission and hence to induce herd protection ^5^.

PCV programs have been designed to provide optimal individual protection of the vaccinees, yet experience in both high income and low income countries indicates that herd immunity, which is generated by reducing carriage and hence transmission of vaccine serotypes in the community, can control vaccine type pneumococcal disease in vaccinated and unvaccinated individuals alike ^6^. Hence it has been proposed that a schedule with a single priming and a booster dose (1p+1) may maintain similar levels of protection for the community by sustaining herd immunity, once circulation of vaccine types has been controlled by the use of higher dose regimens, or by a catch-up campaign ^7^.

Since, it has been shown that indeed a booster schedule with only a single priming dose induces non-inferior post-booster immunogenicity if compared to a 2p+1 schedule, providing some evidence that such reduced-dose schedule could sustain herd protection as hypothesised ^8^. The UK, who had previously spear-headed the reduction from three to two priming doses ^9^, has recently decided to move to a national 1p+1 schedule ^10– 12^.

Using PCV impact on vaccine type carriage as a marker of vaccine type disease ^13^, within this trial we investigate the feasibility of reducing the number of infant doses in the PCV immunization schedule, to make more efficient use of herd immunity in the protection against pneumococcal disease. Rather than observing differences in direct vaccines effects between schedules, our study is designed to directly observe the population level effectiveness of alternative schedules. In order to apply the study outcome in countries using either 3p+0 or 2p+1, we will include both 3p+0 and 2p+1 schedules as control arms in the study. We will also include an investigative 0p+1 arm to test whether population effectiveness can be achieved without a priming dose.

In summary, the following PCV schedules are investigate in this study; (i) 0p+1 – A single dose of PCV at 12 months of age, (ii) 1p+1 – A two-dose schedule of PCV at two and 12 months of age, (iii) 2p+1 – A three-dose schedule of PCV at two, four and 12 months of age, and (iv) 3p+0 – A three-dose schedule of PCV at two, three and four months of age.

(ClinicalTrials.gov Identifier: NCT02961231)

## Methods

### Hypothesis

The cost of PCV use can be greatly reduced by making use of existing herd immunity to protect children against vaccine-type pneumococci. We will reduce the circulation of vaccine-type pneumococci to low levels using a catch-up campaign, after which we will evaluate the ability of a simplified two-dose regimen and an alternative one-dose regimen to prevent the reestablishing of vaccine-type pneumococci.

### Study aims and objectives

To investigate the innovative use of existing and future PCVs to protect communities, in particular in resource-limited settings at lower cost using fewer doses, we will pursue two primary, four secondary and two exploratory objectives as follows:

***Primary objective 1*.** We will evaluate non-inferiority of the 1p+1 schedule compared to a 2p+1 schedule in maintaining control of VT carriage in (a) children aged 4-11 months, *i.e.* after the age of a completed primary series and before age eligibility of a booster dose (this is the population that is most at risk for pneumococcal disease and is likely to receive less direct protection from a reduced primary series), and (b) children aged 14-24 months, *i.e.* after the age where a booster dose is given (this is the beginning of the age period when most intense transmission of pneumococci is believed to occur).

***Primary objective 2*.** We will evaluate the non-inferiority of the 1p+1 schedule compared to a 3p+0 schedule in maintaining control of VT carriage in (a) children aged 4-11 months and (b) children aged 14-24 months

***Secondary objective 1*.** We will evaluate non-inferiority of the 0p+1 schedule compared to a 2p+1 and a 3p+0 schedule respectively in maintaining control of VT carriage in (a) children aged 4-11 months, and (b) children aged 14-24 months.

***Secondary objective 2*.** We will evaluate non-inferiority of the 1p+1 and 0p+1 schedules compared to 2p+1 and 3p+0 schedules in maintaining control of VT carriage in children from the study areas presenting to the local hospital with clinical pneumonia as part of a long-standing enhanced pneumonia surveillance in the study area ^14^.

***Secondary objective 3*.** We will measure the effect of catch-up vaccination in the community on VT carriage four months after the second dose for children eligible for catch-up vaccination.

***Secondary objective 4*.** We will develop mathematical models to predict the impact of 1p+1 and 0p+1 schedules against vaccine type carriage and disease in Nha Trang if introduced simultaneously to all clusters following a catch up campaign, and infer the impact of such schedules in other transmission settings.

***Exploratory objective 1*.** We will measure the impact of PCV vaccination in the four vaccine schedules on pneumonia incidence and the nasopharyngeal serotype distribution and load among pneumonia patients.

***Exploratory objective 2*.** We will measure the impact of PCV, delivered according to the different regimens in this study, on admissions associated with specific respiratory viruses, including influenza and respiratory syncytial virus.

### Study site

The study is conducted in the 27 communes of Nha Trang, central Vietnam which is the capital of Khánh Hòa province. Nha Trang is surrounded by the sea to its East and mountains elsewhere and has about 400,000 inhabitants. Of the 27 communes, eight are classified as rural and 19 as urban. Communes are an administrative unit chosen to be of approximately similar population size and are locally organizing facilities including child-care, schooling and first-line medical care. Nagasaki University has been conducting studies in collaboration with local health authorities in this study site since 2007. PCV10 (Synflorix) has been registered in Vietnam since 2016. However, PCV is yet to be introduced into the national immunization program in Vietnam and private market uptake is minimal.

### Study design

**Figure 1.**
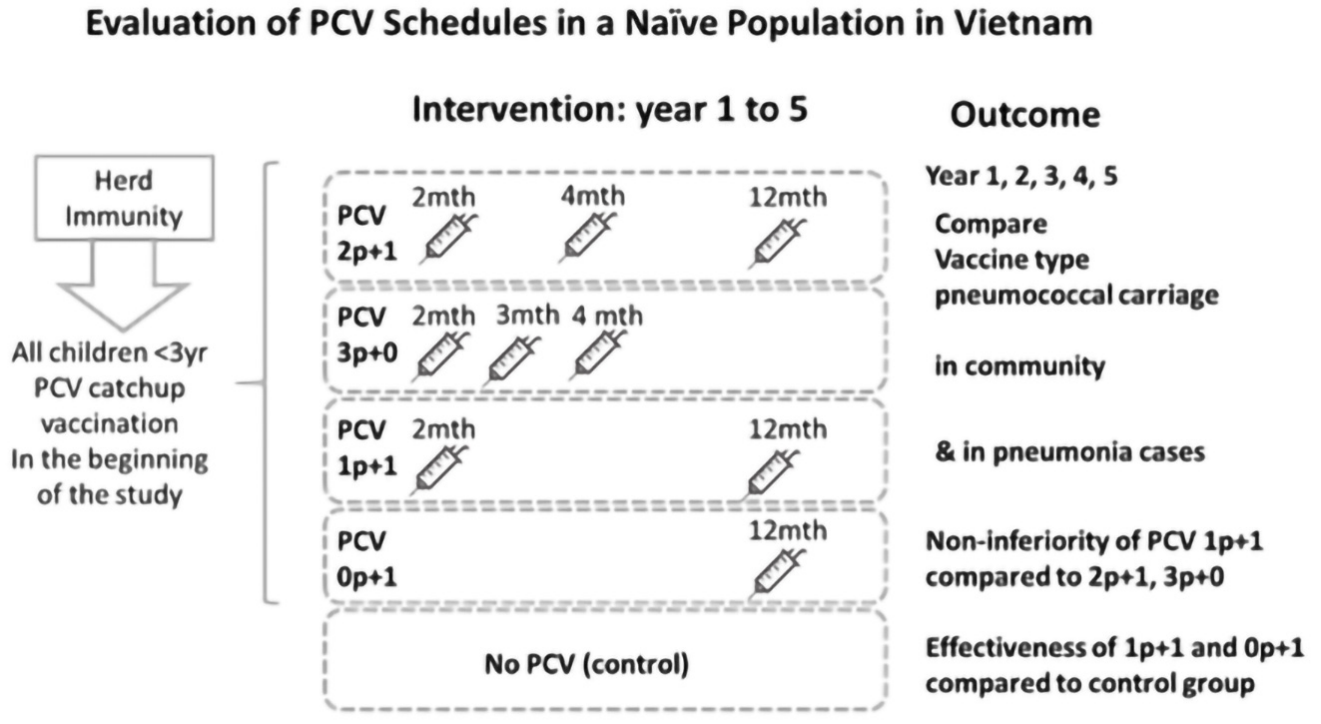
Nha Trang PCV Trial design. The clinical trial design showing the initial catch-up vaccination to all children less than 3 years of age in the PCV intervention arms followed by the randomly designated PCV schedules. The outcome of the study is shown as vaccine type pneumococcal carriage in the community and hospitalized pneumonia cases in the study setting.

We will conduct a Phase IV cluster randomized trial in the 27 communes of Nha Trang city. The four different PCV schedules (1p+1, 0p+1, 2p+1, and 3p+0) form four corresponding intervention arms completed by an informal, non-randomised control arm without PCV use ( Figure 1). The unit of randomization, the clusters, are Nha Trang’s communes. We chose the three clusters that were to remain unvaccinated and randomly allocated the remaining 24 communes into six clusters per intervention arm.

### Selection of PCV for the study

At the time of initiation of the study, PCV10 was registered in Vietnam, while this was not yet the case for PCV13. Therefore, PCV10 is used in the study.

### Background information and sample size calculation

Carriage surveys among healthy children in Nha Trang as well as in hospitalized children admitted with acute respiratory infection found similar serotype coverage for both PCV10 and PCV13; approximately 65% in healthy and 70% in children with acute respiratory illness ^15^. PCV10+6A type pneumococcal prevalence was 33% in healthy infants and 30% healthy toddlers.

### Cluster and sample size calculation

We assumed that (i) the baseline prevalence of carriage of PCV10 serotypes among infants in Nha Trang is 32.5% and varies between communes from 20% to 45% and (ii) that carriage prevalence is reduced to 5% through the catch-up campaign and kept at the same level through routine vaccination under a 2p+1 or 3p+0 schedule.

Nasopharyngeal samples will be collected before the start of PCV vaccination in the site and at 6, 12, 24, 36, 48 and 60 months thereafter from 60 subjects per age groups (4-11mth old children, 14-24mth old children and their mothers) for each of the 27 clusters; adding up to 6,480 swabs per cross-sectional study and 45,360 in total. We assume that 10% of swabs cannot be obtained or cannot be interpreted in the laboratory. We calculated the number of clusters required to test our non-inferiority hypothesis under a type I error probability of 5% and 80% power.

As detailed in Hayes and Moulton ^16^ we calculated that 5.37 clusters are required to detect an increase in VT carriage prevalence among children in the 1p+1 arm compared with the 2p+1 or 3p+0 arms of 5% or more. Hence, six clusters for each intervention arm were included.

### Rationale for inclusion of a single-dose arm

The primary goal of this study is to compare the effect of PCV in a 1p+1 schedule compared to 2p+1 and 3p+0 schedules respectively. However, by extension of the concept of sustaining herd immunity with fewer doses at infancy, a single dose schedule may suffice if the lack of a priming dose does not impair the efficacy of the dose given at 12 months of age. Single PCV doses have been used successfully in toddlers and older children as part of catch-up campaigns to accelerate control of VTs ^17^ but haven’t been investigated as a programmatic choice for routine use yet. This trial set-up posed a unique opportunity to include an additional arm that investigates the effectiveness of a 0p+1 schedule in sustaining population protection against VT carriage.

### Selection of communes for the unvaccinated study arm

The three northernmost communes, Vinh Luong, Vinh Phuong, and Vinh Hoa have been assigned to the unvaccinated control arm based on the geographical background, *i.e.* being somewhat separated from the other Nha Trang communes. Vinh Luong and Vinh Phuong communes are classified as rural communes and Vinh Hoa as an urban commune. Although we will not formally consider these communes as controls for the study as they have not been part of the randomisation, inclusion of unvaccinated communes will provide information on potential year-by-year carriage variations due to introduction of new strains from outside of Nha Trang or from vaccinated communes in Nha Trang.

### Selection of communes for the intervention arms

The remaining 24 communes have been randomized into the four treatment arms. To ensure random but balanced allocation of clusters that were similarly representative of rural and urban communities and that are included in the ongoing hospital-based surveillance, we used automated rejection sampling.

We defined the following set of acceptance criteria:

i) In each arm at least one of the six rural communes must be selected.
ii) In each arm at least three of the 16 communes included in the ongoing pediatric hospital-based surveillance for acute respiratory illnesses must be selected
iii) No two adjacent clusters can be assigned to the same study arm

**Figure 2.**
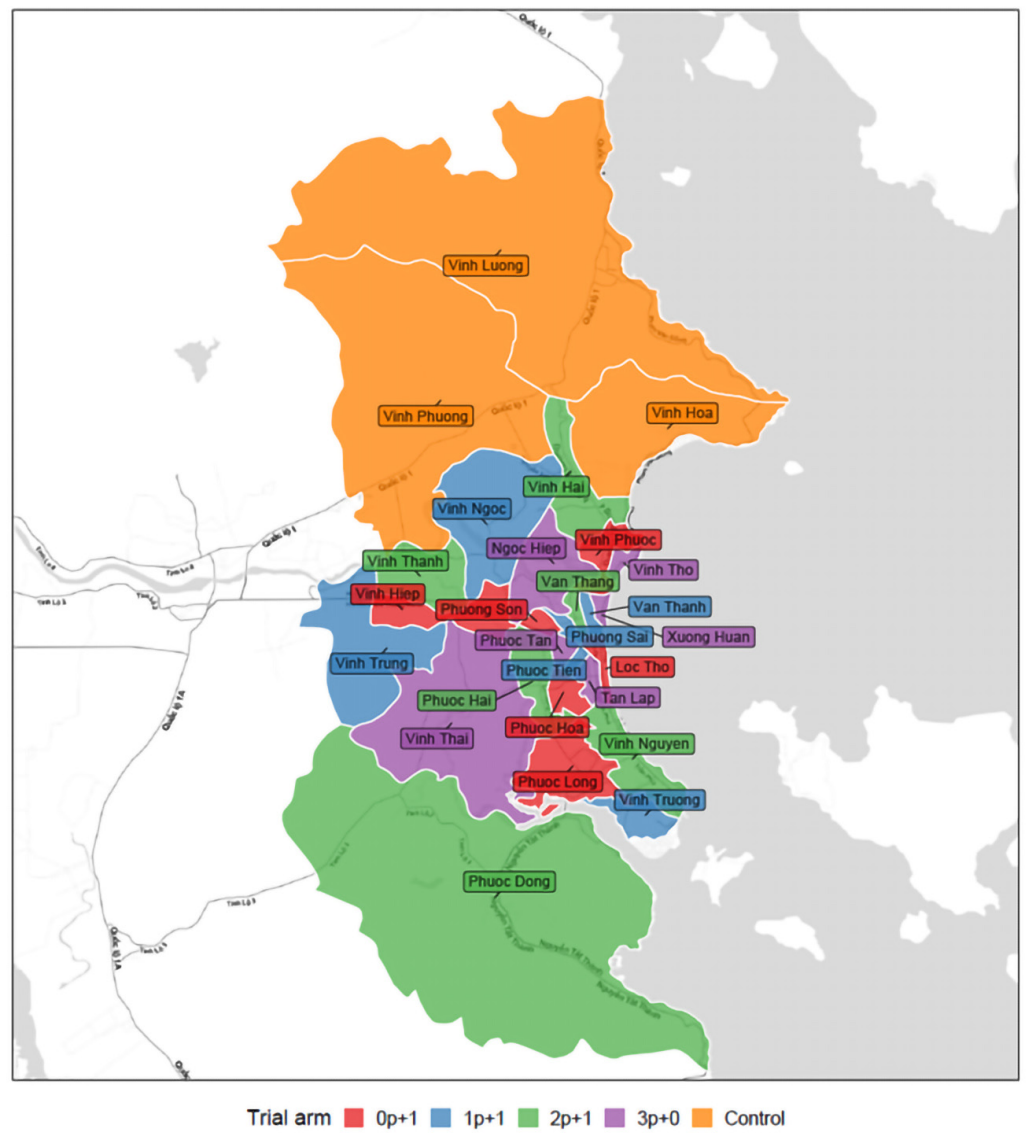
PCV schedule cluster allocation of communes in Nha Trang for each arm. Figure of the 27 communes of Nha Trang city. Cluster/communes receiving different PCV schedules are shown in different colours. Name of the communes are also shown in the figure.

The four color theorem ^18^ indicates that there is at least one possible sampling permutation that fulfills criteria iii). Using the statistical software R ^19^ we developed an algorithm to randomly assign arms to clusters and reject a sample if it violated any of the acceptance criteria above. The program was run until the first sample fulfilled all criteria. In total, 2,793,298 samples were rejected. The final allocation is shown in Figure 2. The rural-urban distribution and current pneumonia surveillance cluster distribution are shown in Table 1.

**Table 1.** PCV schedule, rural-urban and current pneumonia surveillance cluster allocation.

PCV Schedule	0p+1	1p+1	2p+1	3p+0	Unvaccinated
**Setting**					
**Rural**	1	2	2	1	2
**Urban**	5	4	4	5	1
**Pneumonia surveillance**					
**Yes**	3	3	4	3	3
**No**	3	3	2	3	0
**Total**	6	6	6	6	3

### Initial catch-up vaccination

A catch-up vaccination campaign was done in February 2017 before the start of PCV vaccine introduction in all but the unvaccinated three communes. Three doses of PCV10 have been offered to all children between two and six months of age with an interval of two months between the first and second, and seven months between the second and the third doses. A catch-up of two doses of PCV10 has been offered to children between seven and 18 months of age with an interval of two months between the two doses. Children between 19 and 36 months of age have been offered a single dose. This was expected to quickly control circulation of vaccine serotypes. Children with known allergy to PCV10 will be excluded from the vaccination campaign.

### PCV introduction

Children receive PCV based on which cluster they reside in. The detail schedule and time of the vaccination are shown in the Table 2. PCV vaccination started one month after the catch-up vaccination campaign so that all the children under three years of age in the intervention arm communes will receive PCV vaccination. Only those who are known to have allergy to PCV10 will be excluded from the study. Based on census data, we estimated that the annual birth cohort of children in each commune will be about 250 and there will be a total of 6,000 children to be vaccinated per year across the 24 treatment communes. The corresponding number of vaccine doses required is 13,500 per year. Vietnam’s National Immunization Program (NIP) vaccinate DPT-HepB-Hib vaccine at two, three, four months and JE vaccine at 12 months. We will integrate PCV into the same visit as the above NIP vaccines to obtain optimal coverage.

**Table 2.** Different PCV vaccination schedules and timing of the vaccination.

Schedule	0p+1	1p+1	2p+1	3p+0	Unvaccinated
**2 months**		X	X	X	
**3 months**				X	
**4 months**			X	X	
**12 months**	X	X	X		

### Cross-sectional carriage surveys

We will conduct cross-sectional nasopharyngeal carriage surveys before the catchup vaccination campaign (baseline carriage survey), four months after the second dose of catch-up vaccination (post-catchup carriage survey), and annually during the same month that the baseline survey was conducted in. In each carriage survey, 60 children four to 11 months old, 60 children 14 to 24 months old and their mothers (120) in each of the 27 study clusters will be enrolled and screened for the presence of vaccine-serotype carriage. The census database and regularly updated EPI vaccination list of children are used to randomly select study subjects. The data manager from NU will create a random list based on a list of eligible children using Stata software version 14. Informed consent will be obtained from mothers or guardians of the child before enrolment by medical doctors or health staff at commune health centers. The baseline carriage survey will reveal the background *S. pneumoniae* carriage patterns while the second carriage survey after the catchup vaccination will reveal the effect of catchup vaccination and initial vaccine introduction in the community. The subsequent annual carriage surveys will reveal the *S. pneumoniae* carriage pattern among clusters receiving different PCV vaccination schedules.

### Sample collection and testing

Nasopharyngeal samples will be collected from the study participants. The initial screening test will be conducted locally at the Pasteur Institute in Nha Trang. DNA will be extracted from the nasopharyngeal samples and screened for the *S. pneumoniae* lytA gene using real-time PCR. The positive samples will be cultured, DNA extracted and then the DNA samples will be transported to MCRI for serotype determination by Microarray assay, the most sensitive of serotyping methods ^20^. All the data will be double-entered and checked before the data analysis. Data managers from NIHE and NU will manage the database, and only the data without personal information will be shared with other main investigators from the London School of Health and Tropical Medicine (LSHTM) and Murdoch Children's Research Institute (MCRI) for data analysis. In accordance with the ethical approval, the residual samples will be stored for five years after completion of the study for future confirmatory testing. Clinical trial insurance contract has been obtained to cover the AE, SAE treatment cost and compensatory payments.

### Patient and Public Involvement

The study subjects were less than 24 month-old children, eligible for routine vaccination in the community and they were not involved in the development of research question, outcome measures, the design of the study. Fieldworkers from the commune health centers conduct the home visit, explained the study and requested informed consent from the parents or guardian of the subjects to participate in the study. The results of the study will be shared with the local health authorities and the local health authorities will disseminate the study results to the parents and guardian of the participants. The parents or guardian of the children participating in the study were asked to report any discomfort or side effect of the PCV10 vaccination and nasopharyngeal sample collection during the carriage surveys. 

### Approach and data analysis

The comparison of carriage prevalence in the intervention (1p+1 or 0p+1) clusters and the gold standard (2p+1 or 3p+0) clusters respectively will allow age-group specific assessment of non-inferiority of the 1p+1 and 0p+1 schedules if compared with the 2p+1 or 3p+0 schedules respectively in regard to protection against vaccine type carriage. The data will be analysed single-blindedly through an intention-to-treat analysis as this yields the most conservative effect that could be observed in a real-world setting. An absolute difference in prevalence of <5% will be used as the non-inferiority margin (see sample size calculations). As the primary analysis we will use a Chi-squared test of a difference in two proportions to estimate the treatment effect ( *i.e.* the difference between 1p+1 and 2p+1 arm VT prevalence in infants) following the approach detailed in Hayes and Moulton ^16^ for trials with fewer than 15 clusters per arm. The upper limit of the two-sided 95% confidence interval of the treatment effect not exceeding the specified non-inferiority margin of 5% will indicate non-inferiority.

***Mathematical modelling analysis*.** We will use data collected on age-specific VT and NVT prevalence to fit a dynamic age-structured meta-population model of pneumococcal transmission to changes in pneumococcal carriage prevalence over the study period in the 27 study communes. We will model the transmission dynamics of each of the five trial arms separately whilst also allowing for some degree of population movement between the arms to reflect the general dynamic behavior of the population. Incorporating movement will account for the possibility of spill-over effects between the different arms of the trial. Parameter estimation will be performed using adaptive Markov Chain Monte Carlo; we will estimate nine key parameters within the model from the collected data. These will be: age-group specific transmission rate for three key age groups for VT and NVTs (six parameters), the level of protection within each arm given by 1p+0 and 1p+1 (two parameters) and the rate of waning for the protection given by the 1p+1 dosing schedule. Chain convergence will be assessed through the use of the Gelman-Rubin statistic and efficient exploration of the posterior sample space will be confirmed by calculating the effective sample size. The estimated model parameters will then be used to predict the impact of PCV in a scenario where PCV had been introduced using the 1p+1 or 0p+1 schedules in all 27 communes. We will also use this framework to infer the impact of continuation with a 1p+1 or 0p+1 schedules in other transmission settings where PCVs have been in use and where high quality allows model calibration (secondary objective 4).

### Hospital pneumonia surveillance and severe adverse events monitoring

To address exploratory objective 1 and 2, we will monitor the incidence of pneumonia in the study areas, as a continuation of an ongoing population-based pneumonia surveillance project that has been in place in the area since 2007 ^21^. Nasopharyngeal swabs will be taken from children attending to Kanh Hoa Province General Hospital (KHGH), the only public hospital in Nha Trang, with signs of clinically or radiographically confirmed pneumonia and coming from the study areas as an additional means of monitoring vaccine-type carriage in the study areas. This will enable us to evaluate this strategy of sampling for monitoring potential re-emergence of VT carriage in communities using the reduced schedule approach.

### Safety monitoring

The trial meets the ethical and regulatory requirements to monitor serious adverse events (SAE) in line with standards of good clinical practice. A SAE was defined as any event requiring hospitalization or resulting in death. Using the ongoing surveillance for acute respiratory illnesses at KHGH, we will monitor SAE potentially related to PCV vaccination. Any SAE such as hospitalization or death of a child within one month after receiving PCV will be investigated for its association with PCV vaccination. This process is similarly conducted for routine EPI vaccines as well. The Community Health Centre (CHC), local polyclinics and the Preventive Medicine Department of Kanh Hoa Health service in Nha Trang will support and collaborate on PCV related SAE monitoring. These health care institutions are also involved in the SAE monitoring related to EPI vaccination program in Vietnam and the safety monitoring meetings will be conducted every three-to-four months. If dangerous SAE are reported, the safety monitoring team can instruct to stop the study and report to MOH.

## Trial status

In October 2016 the baseline carriage study was conducted, followed by PCV introduction and a catch-up campaign in accordance with randomization in January and February 2017. The year 5 post introduction survey, expected to take place in October 2021, was postponed to 2022 July due to the COVID-19 outbreak situation in Vietnam and analyses for primary endpoints are expected to be completed by first quarter of 2023.

### Ethics approval and consent to participate

Ethical approval was obtained by Ministry of Health, Vietnam (4875/QD-BYT), and Nagasaki University (15120149).

***Strengths and limitations of this study.*** This study is designed to address whether a reduced PCV dosing schedule of one primary and one booster (1p+1) can be applied in a population with herd immunity against vaccine type PCV.

This study includes a PCV catch-up vaccination followed by a cluster randomized trial with four PCV intervention arms (1p+1, 0p+1, 2p+1, 3p+0) and three unvaccinated clusters in a PCV naïve population.

The primary outcome is the non-inferiority of vaccine type pneumococcal carriage among children in 1p+1 arm cluster compared to WHO recommended 2p+1 and 3p+0 arms clusters.

Limitations of the study include limited power for secondary outcomes.

## Figures and Tables

**Annex 1.** Study site background information 27 communes of Nha Trang city.

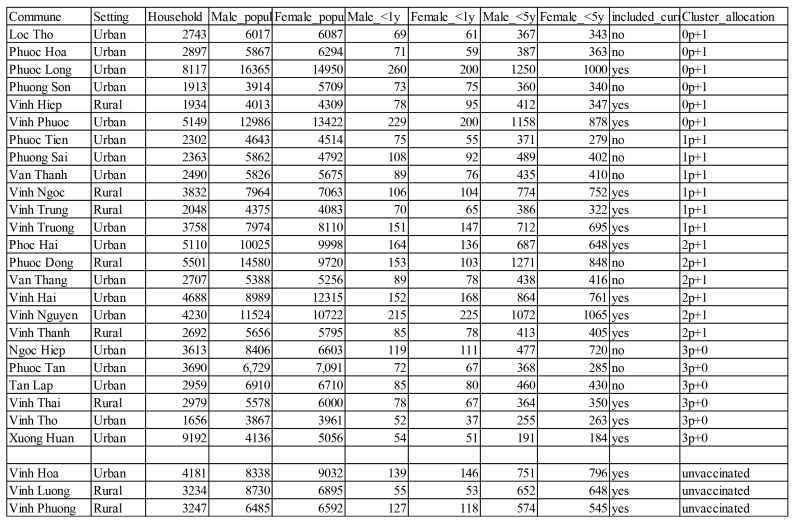

## Data Availability

No data are associated with this article.
