# Supplementary material for: Evaluation of the effect of reduced-dose pneumococcal conjugate vaccine schedules on vaccine serotype carriage in children and their caretakers in a naïve population in Vietnam: Protocol for a cluster randomized non-inferiority trial
Source: Gates Open Res. 2023 Jul 20;7:110. [Version 1] doi: 10.12688/gatesopenres.14742.1 (PMC10539539; doi:10.12688/gatesopenres.14742.1)
Supplement: Peer review history

# Reviewer report: Reviewer response for version 1

**DOI:** 10.21956/gatesopenres.16068.r34870

**Published:** 2023-09-28

**Authors:** Stephanie Perniciaro

**Affiliations:** 1 Epidemiology of Microbial Diseases, Yale University, New Haven, Connecticut, USA

This article describes a hotly-anticipated trial of pneumococcal conjugate vaccine schedules taking place in Vietnam. The optimal schedule for infants, balancing control of vaccine-serotype pneumococcal disease and the high cost of pneumococcal conjugate vaccines, has been a subject of extensive debate in the pneumococcal and vaccinology communities in recent years. The design of this trial seeks to directly compare vaccine serotype carriage in populations receiving either (presumed) 0, 1, 2, or 3 doses of pneumococcal conjugate vaccines during infancy. A catch-up campaign for children 3 and under was implemented following randomization but prior to beginning the experimental vaccine schedules. The COVID-19 pandemic interrupted the expected sampling intervals following introduction of the experimental schedules. 

 Like many in the field, I am intensely curious to see the results of this trial, in particular, with regard to the conduct of the trial (the uptake level for the catchup program, the compliance with the vaccine schedule in different randomized groups, and the follow-up rates of sampling) and, of course, the results. How many doses of pneumococcal conjugate vaccine are sufficient to control the circulation of vaccine-serotype carriage in a naive population? How long does protection against vaccine serotype carriage from the booster dose last? What happens in the unvaccinated regions? What is carriage like in adults? This will be a provocative and enlightening trial, and I will look forward to seeing the results.

Is the study design appropriate for the research question?

Yes

Is the rationale for, and objectives of, the study clearly described?

Yes

Are sufficient details of the methods provided to allow replication by others?

Yes

Are the datasets clearly presented in a useable and accessible format?

Not applicable

Reviewer Expertise:

pneumococcal epidemiology, pneumococcal vaccine effectiveness, pneumococcal vaccine schedules

I confirm that I have read this submission and believe that I have an appropriate level of expertise to confirm that it is of an acceptable scientific standard.

# Reviewer report: Reviewer response for version 1

**DOI:** 10.21956/gatesopenres.16068.r34872

**Published:** 2023-09-25

**Authors:** Alamgir Kabir

**Affiliations:** 1 Centre for Primary Health Care and Equity, University of New South Wales, New South Wales, Australia

The study titled "Evaluation of the effect of reduced-dose pneumococcal conjugate vaccine schedules on vaccine serotype carriage in children and their caretakers in a naïve population in Vietnam: Protocol for a cluster randomized non-inferiority trial" by Yoshida et al. (2023) holds significant importance in evaluating the efficacy of reduced-dose pneumococcal vaccination regimens. If the findings of this study confirm that the reduced-dose schedule is as effective as the WHO-recommended higher-dose schedule, it could have profound implications for the global control of pneumococcal disease, especially in the low- and middle-income countries (LMICs). Given the high cost of pneumococcal vaccines, a reduced-dose schedule could yield substantial cost savings and play a pivotal role in sustaining pneumococcal vaccinations programs even with no or reduced support from the organisation like GAVI especially in the LMICs.

 Furthermore, this research aims to address a crucial question: whether administering a single vaccine dose at 12 months of age, as part of the catch-up schedule, is adequate for conferring protection against pneumococcal disease in later age. While the recommendation for this one-dose catch-up schedule exists, empirical evidence to support it is currently lacking. Therefore, the outcomes of this study have the potential to significantly impact global efforts to prevent the burden of pneumococcal disease. Considering all those discussed above, I strongly recommend to public this study.

 This is a very well-written manuscript. However, I have some minor comments for the authors for consideration to improve the clarity. Below are my specific comments.

 Abstract:

1. The schedules of the vaccine, such as 3p+0, 2p+1 has not been clearly defined in the abstract.

Introduction:

1. Again, vaccination schedules have not been defined adequately. However, they have been clearly described in Figure 1, so it is good to define clearly in the introduction and refer Figure 1.
2. This study is going to evaluate the effectiveness of a single-dose pneumococcal vaccination at 12 months of age, as part of the catch-up schedule. I think, it would be good to provide some data if available on the effectiveness of 0p+1 dose schedule. If there are no studies evaluating the effectiveness of 0p+1 schedule, I think, that would be worth mentioning in the introduction.

Methods:

1. Primary objective: It is good to define the non-inferiority, i.e. non-inferiority margin.
2. Study endpoints- I think it is also good to provide a justification of using VT carriage as a study endpoint instead of the more specific outcome, VT invasive pneumococcal disease.
3. Secondary objective 2: In the previous objectives, the authors clearly mentioned that the efficacy of the schedule will be measured for two different age groups, but for secondary objective 2, they have not mentioned whether they will evaluate the efficacy for different age groups or for all children. If there is no budget constraint, I would recommend to do a vaccine-type IPD surveillance to measure the effectiveness of the vaccine against the more specific outcome.
4. Secondary objective 4: I think the analysis for objective 4 is conditional on the other previous objectives. If the reduced-dose schedules are not effective at the desired level, it is not worth having a predictive model for the reduced-dose schedule.
5. Sample size calculations: This section has not been clearly written. For example, what was the non-inferiority margin and how the design effect has been adjusted. As this paper will be cited by other papers arising from this study, I would recommend to write the sample size calculation section in more detail.
6. Approaches and data analysis: It seems to me that the statistical analysis section is inadequate. The study has several objectives, but the authors only have written the statistical analysis methods for the primary objectives not for the secondary ones. For example, the statistical analysis method might be different for secondary objective 2 as they will have a passive surveillance data for the study endpoints. Hazard ratio (Cox regression model) can be used to measure the effectiveness of the new schedules for this objective.

Finally, I would like to thank all the authors for their effort in addressing this important and timely research question.

Is the study design appropriate for the research question?

Yes

Is the rationale for, and objectives of, the study clearly described?

Yes

Are sufficient details of the methods provided to allow replication by others?

Partly

Are the datasets clearly presented in a useable and accessible format?

Not applicable

Reviewer Expertise:

Infectious disease, vaccine evaluation, biostatistics

I confirm that I have read this submission and believe that I have an appropriate level of expertise to confirm that it is of an acceptable scientific standard, however I have significant reservations, as outlined above.

# Reviewer report: Reviewer response for version 1

**DOI:** 10.21956/gatesopenres.16068.r34724

**Published:** 2023-08-25

**Authors:** Anand Kawade

**Affiliations:** 1 Vadu Rural Health Program, King Edward Memorial Hospital Research Centre (KEMHRC), Pune, Maharashtra, India

**General comments:**

 At the outset I would like to congratulate the authors for taking up this important gap to explore. There has been a constant trade-off between higher prizes of PCV and reduction in doses or schedule, especially for programmatic consideration in LMIC which carries the highest burden of IPD. This seems to be completely new strategy for PCV program for those countries who can’t afford and sustain PCV in regular program. Once the pneumococcal transmission is controlled to a very low level by catch-up vaccination, we can maintain the level of herd protection by either of 1p+1 (younger age) or 0p+-1 (elder age) PCV schedule. This could be a cost effective option. Observing the population level effectiveness of alternative schedules will be of great help in deciding the programmatic inclusion of PCV.

 Overall, this is a greatly written protocol where all the aspects of design and conduct of clinical trials have been considered.

**Specific comments:**

**Abstract**:

Introduction:

 Sentence “three doses in infancy with Pentavalent vaccine (3p+0)” might be misinterpreted by readers considering P as abbreviation for “pentavalent”. We know that 3p+0 is “three primary doses with no booster”. Therefore, to avoid confusion, can we say, “three primary doses in infancy with Pentavalent vaccine”?

**Main Text:**

Introduction:

 I suggest adding 1p+1 evidence of noninferiority of schedule in VT carriage reduction from other countries South Africa, Cuba, India to make your case stronger.

Hypothesis:

 Can you rewrite in the hypothesis format? Like: ‘We hypothesized that the reduced dose schedules of PCV; two doses and single dose schedules, are similarly effective in maintaining herd protection.”

Objectives: Needs some clarity.

Primary objective:

- Your primary objective is comparison of schedule in maintaining control of VT carriage. So, it will be prudent if you could define “maintaining control of VT carriage” (e.g., Like 5 % with +/- 5% margin etc.). Or, what do you mean by control of VT carriage? And whether this estimation will be population based? And is this same for both the age groups? (4-11 months and above 11 months).
- Secondary objective 1: Are both the schedules combined or analyzed separately?
- Secondary objective 2: Are the schedules compared for effectiveness in preventing clinical pneumonia? Are the schedules combined or analyzed separately?
- Secondary objective 3: If we are taking samples four months after second dose then are we excluding 3+0 recipients?
- Exploratory 1: clinical outcome, will it be a part of “pneumonia surveillance or this protocol?
- Exploratory 2: Impact on RSV & influenza : are we considering only hospital admission or less severe infection also?
- For both exploratory objectives are you taking different bio-sampling (for pneumococci & for viral) for clinical care or research purpose? Is separate consent needed?

Population:

- Please enumerate common serotypes at baseline in infants & in under 5 children.
- Effect on Caretaker: Although it is mentioned in the title, objective & endpoints related to caretaker are not found. I suggest adding.

Design:

- Cluster and Sample size calculation:
  This section says, “We assumed that (i) the baseline prevalence of carriage of PCV10 serotypes among infants in Nha Trang is 32.5% and varies between communes from 20% to 45% and (ii) that carriage prevalence is reduced to 5% through the catch- up campaign and kept at the same level through routine vaccination under a 2p+1 or 3p+0 schedule.”
  If I understood correctly, this means you expect that the pre-vaccination (catch-up) carriage prevalence will be reduced from 20-45% to 5%. This percentage reduction will be 75% for baseline carriage of 20% (20-5*100/20) and will be almost 90% [88.8%] for baseline carriage of 45% (45-5*100/45). I am not sure whether this would happen in short time span of 4 months. Request to explain.   
- Sample size is based on increase in VT carriage prevalence among children in the 1p+1 arm compared with the 2p+1 or 3p+0 arms of 5% or more.
  However, this needs to be aligned with the objective (instead of “maintaining the control”).
- In mathematical modelling section you have mentioned “three key groups” (page 7, right para, line 3). Which are they?

Is the study design appropriate for the research question?

Yes

Is the rationale for, and objectives of, the study clearly described?

Yes

Are sufficient details of the methods provided to allow replication by others?

Yes

Are the datasets clearly presented in a useable and accessible format?

Not applicable

Reviewer Expertise:

Vaccine, infectious disease, paediatrics, public health

I confirm that I have read this submission and believe that I have an appropriate level of expertise to confirm that it is of an acceptable scientific standard.
